# Population dynamics, delta vulnerability and environmental change: comparison of the Mekong, Ganges–Brahmaputra and Amazon delta regions

**DOI:** 10.1007/s11625-016-0372-6

**Published:** 2016-05-26

**Authors:** Sylvia Szabo, Eduardo Brondizio, Fabrice G. Renaud, Scott Hetrick, Robert J. Nicholls, Zoe Matthews, Zachary Tessler, Alejandro Tejedor, Zita Sebesvari, Efi Foufoula-Georgiou, Sandra da Costa, John A. Dearing

**Affiliations:** 1 Division of Social Statistics and Demography, University of Southampton, Southampton, UK; 2 Department of Anthropology, The Anthropological Center for Training and Research on Global Environmental Change and the Ostrom Workshop-Indiana University, Bloomington, USA; 3 United Nations University, Institute for Environment and Human Security, Bonn, Germany; 4 Engineering and the Environment, University of Southampton, Southampton, UK; 5 Environmental CrossRoads Initiative, City University of New York, New York, USA; 6 Civil, Environmental and Geo-Engineering and National Center for Earth-surface Dynamics, University of Minnesota, Minneapolis, USA; 7 Instituto de Pesquisa e Desenvolvimento, Universidade do Vale do Paraíba (UNIVAP), São José dos Campos, Brazil; 8 Geography and Environment, University of Southampton, Southampton, UK

**Keywords:** Population change, Delta vulnerability, Mekong delta, Amazon delta, Ganges–Brahmaputra delta

## Abstract

Tropical delta regions are at risk of multiple threats including relative sea level rise and human alterations, making them more and more vulnerable to extreme floods, storms, surges, salinity intrusion, and other hazards which could also increase in magnitude and frequency with a changing climate. Given the environmental vulnerability of tropical deltas, understanding the interlinkages between population dynamics and environmental change in these regions is crucial for ensuring efficient policy planning and progress toward social and ecological sustainability. Here, we provide an overview of population trends and dynamics in the Ganges–Brahmaputra, Mekong and Amazon deltas. Using multiple data sources, including census data and Demographic and Health Surveys, a discussion regarding the components of population change is undertaken in the context of environmental factors affecting the demographic landscape of the three delta regions. We find that the demographic trends in all cases are broadly reflective of national trends, although important differences exist within and across the study areas. Moreover, all three delta regions have been experiencing shifts in population structures resulting in aging populations, the latter being most rapid in the Mekong delta. The environmental impacts on the different components of population change are important, and more extensive research is required to effectively quantify the underlying relationships. The paper concludes by discussing selected policy implications in the context of sustainable development of delta regions and beyond.

## Introduction

Delta regions constitute dynamic ecological and social environments and are often major contributors to national economies. While overall, deltas account for only 1 % of global land area, they are home to more than a half billion people, or ca. 7 % of world population (Ericson et al. 2006). Given the particular environmental risks faced by tropical deltas and accounting for interlinkages between demographic and environmental factors, it is crucial to analyze population dynamics in delta environments to inform planning and policy making. The Millennium Ecosystem Assessment (2005) showed that beyond provisioning ecosystem services such as food, water, fiber and fuel, regulating and supporting ecosystem services also influences various aspects of human development, such as health and income, and long-term sustainability of agriculture and natural resources. Trends in human wellbeing, however, cannot be fully understood without considering the specific demographic context, including evolving population structures by age and sex, and changes in the dominant demographic drivers of fertility, mortality and migration.

Understanding population trends and dynamics in deltaic regions is especially important in the context of global environmental change which is expected to exacerbate the existing threats to livelihoods through e.g., sea level rise, land subsidence, increased storminess, flooding, and salinity intrusion (Dun 2011; Nicholls 2011; Szabo et al. 2015a, b, c; Wong et al. 2014; World Bank 2000). In this context, the present study examines population dynamics (population growth, fertility, mortality, and migration) in three selected tropical deltas. It draws on the theory of demographic transition and the literature conceptualizing the interlinkages between population and environment (de Sherbinin et al. 2007; Hummel et al. 2012). According to fundamental demographic theory (Notestein 1945; Dyson 2011; Dyson 1998), it is common for countries to experience concurrent falling mortality and fertility levels as they progress through the ‘demographic transition’. This is a process through which a country evolves from high to low levels of mortality and fertility, usually associated with increasing longevity. With regard to interlinkages between population and environment, specific components of demographic change can also be influenced by the quality of the biophysical environment, environmental hazards and creeping processes, such as salinity intrusion and arsenic contamination of water and soil resources.

The present study focuses on three specific delta regions, i.e., the Ganges–Brahmaputra delta in Bangladesh (GBD), Mekong delta in Vietnam and the Amazon delta in Brazil. These deltas were selected as they are each locally and globally significant and encompass a range of biophysical and social conditions. Population size, the rate of population growth and population distribution constitute crucial factors which are affecting and affected by natural habitat. While a relatively large body of literature examined the interlinkages between population growth and environment (de Sherbinin et al. 2007; Hummel et al. 2012; Lutz et al. 2002), there is limited consideration of the dynamics of population change in delta regions. Yet, approximately 10 % of the world’s population live in areas lower than 10 m above sea level, and the population in these low-lying coastal areas is projected to grow in all continents, especially in the developing world (McGranahan et al. 2007; Neumann et al. 2015). By providing a rigorous demographic overview of three delta regions, the study not only contributes to the literature on delta regions, but also considers policy implications for sustainable development.

The next section describes the data sources and methods used in this study. In “Environmental context for delta vulnerability”, we discuss the environmental context for vulnerability of tropical deltas using the examples of the three case studies, i.e., the Ganges–Brahmaputra, the Mekong and the Amazon delta. In “Feedbacks between population dynamics and environmental change”, we propose an original conceptual framework illustrating the feedbacks between population and environment with a specific focus on delta regions. “Population growth and structure” summarizes the recent trends in population growth across the study areas, while “Components of population change” offers an analysis and discussion of the key components of population change in the three delta regions. The final section summarizes the key arguments and discusses the main policy implications in the context of the projected population dynamics and the current and expected impacts of environment and climate change on the delta regions and delta populations.

## Materials and methods

### Study areas

**Fig. 1:**
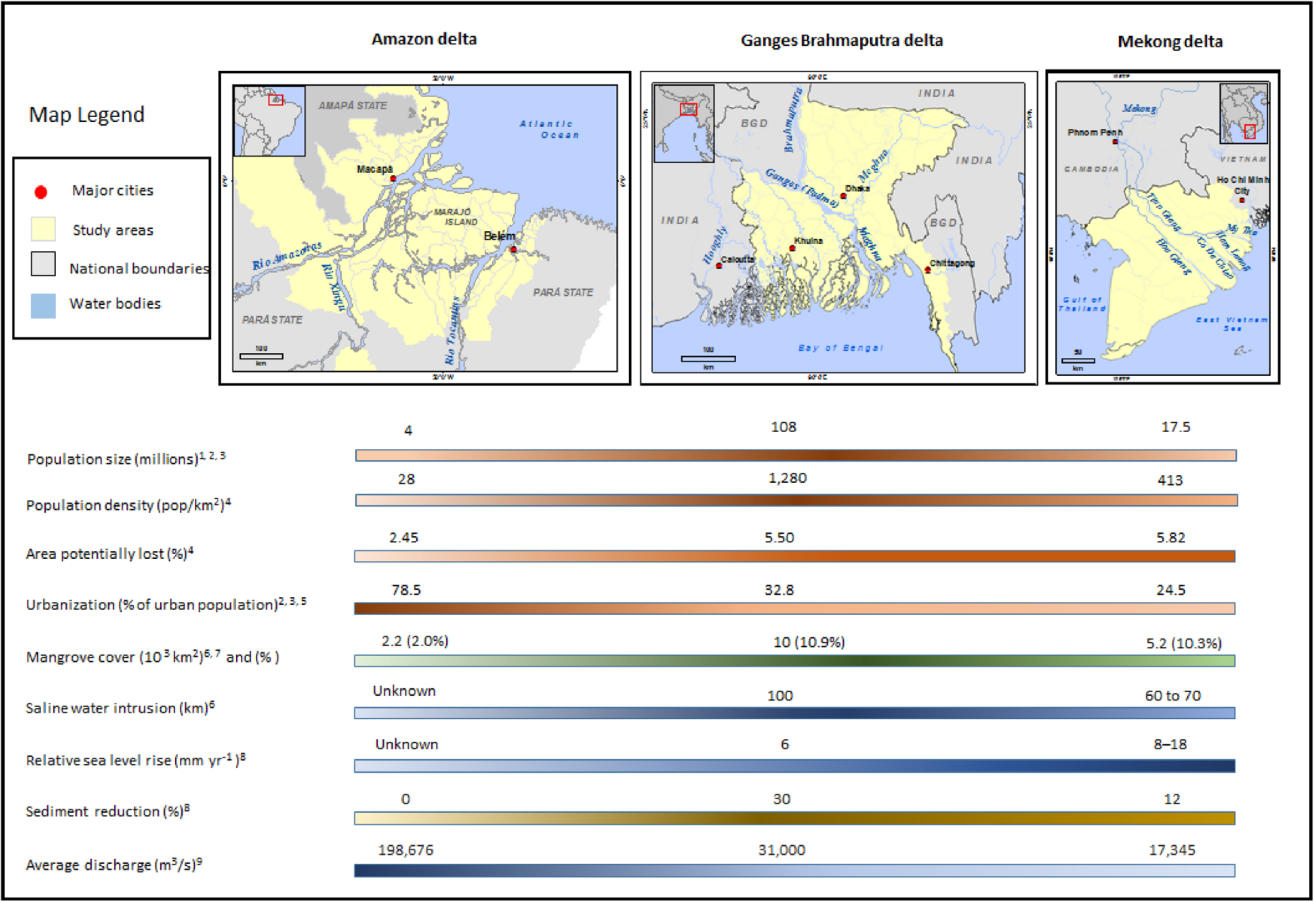
Population-environment gradients in the study areas. Note *1* Bangladesh Population and Housing Census (2001, 2001 and 1991); *2* Brazilian Institute of Geography and Statistics (IBGE 2010); *3* 2009 Vietnam Population and Housing Census; *4* Ericson et al. (2006); *5* World Development Indicators (WDI), World Bank; *6* Parry et al. (2007); *7* Giri et al. (2011); *8* Syvitski et al. (2009); *9* Syvitski and Saito (2007)

The study areas (Fig. 1) encompass three deltaic systems, all located in developing/transition countries. For the purposes of data collection, we have defined the spatial extent of each delta primarily by the area downstream of the first distributary as mapped by the Shuttle Radar Topography Mission.

For the Ganges–Brahmaputra delta (GBD), the altitude of the first distributary (the Hoogli river) at the Farraka Barrage is about 18–20 m asl. Within Bangladesh, this contour encloses over 45 districts in whole division areas of Khulna, Barisal, Dhaka, Sylhet but most of Chittagong division. Chittagong division excludes the districts of Khagrachari, Rangamati and Bandarban. This is an environmentally vulnerable region suffering both from the consequences of rapid-onset hazards (e.g. cyclones) (Kay et al. 2015) and creeping processes, such as salinity intrusion (Clarke et al. 2015), arsenic contamination of groundwater (Abedin et al. 2012; Edmunds et al. 2015) and subsidence (Brown and Nicholls 2015). There is extensive evidence that multiple stressors associated with environmental change place increasing strains on the livelihoods of populations in the region. In particular, food security has emerged as a key developmental concern (Faisal and Parveen 2004; MEF 2009).

For the Mekong, the first distributary point at Phnom Penh lies at about 7–9 m asl which delineates a delta mainly in Vietnam with a small part in Cambodia. The Vietnamese portion of the Mekong delta region (thereafter: the Mekong delta) covers 13 provinces and excludes Ho Chi Min City. As with the GBD, the Mekong delta is highly vulnerable to adverse environmental events, in particular, flooding and salinity intrusion. While it has been recognized that fluvial floods can bring benefits for the economy, as they convey sediment and benefit fisheries (Tri et al. 2013), flooding can also have a disastrous effect on households’ livelihoods. Since 2000, the region experienced three major floods (2000, 2001 and 2002); the first affecting approximately 11 million people. As a result of this flood, 800 × 10^3^ dwellings were inundated, and 55,123 ha of rice crops destroyed (Nguyen and James 2013). Extreme weather events will continue to occur in the region and may occur more frequently (Dun 2011). In addition, climate change is likely to increase not only the risk of flooding, but is also associated with relative sea level rise, salinity intrusion and changes in temperature and rainfall patterns (Dang et al. 2014; Nguyen and James 2013).

Finally, for the Amazon delta (Brazil), we combined the parameters provided by Ericson et al. (2006), who used a 5 km buffer zone around the coastline intersecting with the first distributary, and the limits of municipalities intersecting this buffer zone to define our study area. In terms of administrative boundaries, the study area comprises 50 municipalities across the Pará and Amapá states in the North region of Brazil (or approximately 6 % of the legal Brazilian Amazon in terms of total territory). In 2010, our study area concentrated approximately 16.5 % of the total population of this legal Amazon and 18 % of its urban population (IBGE 2010). While other parts of the Brazilian Amazon have undergone significant environmental change during the last three decades, the delta region has seen relatively lower levels of environmental degradation. However, the region has experienced rapid urbanization, so there are local hotspots of environmental change and a growing economy based on forest products and agroforestry (Brondizio et al. 2013). Most urban areas in the region lack basic sanitation and other infrastructure and public services, which along with some of the highest poverty rates in Brazil create vulnerable conditions for a significant segment of the population (this issue). On the other hand, farmers in the region are reporting increasing tidal flooding and changing salinity in coastal ecosystems, but these changes have not been systematically documented.

Figure 1 illustrates the key delta relevant population-environment gradients and socio-environmental characteristics of the three study areas. Gradient bars, associated with variables are shaded to represent the respective “values” of each variable for each study area. Darker shades represent higher values. Population size and population density are greatest in the GBD where the total population exceeded 108 million, and the population density in the study area is approximately 1280 people per km^2^ (Ericson et al. 2006). The proportion of delta population at risk is highest in the Mekong delta region, and so is the proportion of delta area potentially lost by 2050 (Ericson et al. 2006). It should, however, be noted that, because of the population size of the GDB, in terms of absolute numbers, the greatest impact on population loss is expected to take place in this delta region (Ericson et al. 2006). Among the three delta regions, the Amazon delta has the highest proportion of urban population, while the Mekong delta is least urbanized.

### Data

Our paper draws on a number of secondary macro and micro level data sources to overview and analyze the three deltas discussed above. More specifically, for Bangladesh, the data used include the 2010 household income and expenditure survey (HIES) conducted by the Bangladesh Bureau of Statistics (BBS 2011a), Demographic and Health Surveys (DHS) (Mitra et al. 1994; NIPORT, Mitra and Associates and ICF International 2013; NIPORT, Mitra and Associates and Macro International 2009; NIPORT, Mitra and Associates and ORCM 2001) as well as census data. Similarly, for Vietnam, we used the Vietnamese Living Standards Survey (VLSS), census data and Demographic and Health Surveys. Finally, for the Amazon delta, we used census data from the Brazilian Institute of Geography and Statistics (IBGE) and demographic data and human development indices compiled by The Institute of Applied Economic Research (IPEA) of Brazil (IBGE 2010; IPEA 2010). Data are aggregated at the municipal and census sector levels for 1991, 2000 and 2010 in all three delta regions. Table 1 summarizes the demographic data sources used in the analysis.

**Table 1:** Sources of demographic data

Delta region	Data source
Ganges–Brahmaputra	Bangladesh Demographic and Health Surveys (DHS), 2010 household income and expenditure survey (HIES), Bangladesh Population and Housing Census (2001, 2001 and 1991)
Mekong	Vietnamese Living Standards Survey (VLSS), Vietnam Demographic and Health Surveys (DHS), 2009 Vietnam Population and Housing Census, online data repository developed by the General Statistics Office of Vietnam
Amazon	Brazilian Institute of Geography and Statistics (IBGE 2010, 2014) Institute of Applied Economic Research (IPEA 2010)
Cross-cutting	World Population Prospects (United Nations, 2012)

## Environmental context for delta vulnerability

Sea level rise associated with global climate change is rightly considered a major factor in setting the vulnerability and sustainability of coastal systems (Brakenridge et al. 2013). Rising seas increase the risk of flooding due to coastal storms (Balica et al. 2012) and increase rates of coastal wetland loss (Nicholls 2004; Spencer et al. 2016). In deltas, sea level rise is exacerbated by land subsidence, resulting in relative sea level rise (RSLR) rates several orders of magnitude greater than eustatic sea level rise rates alone (Ericson et al. 2006; Syvitski et al. 2009). As environments of active sedimentation, deltas rely on delivery and deposition of new sediment from the upstream catchment area to offset natural sediment compaction (Syvitski and Saito 2007). Human activity on the delta can accelerate these natural rates through urban development (Mazzotti et al. 2009), groundwater extraction (Higgins et al. 2013) and hydrocarbon extraction (Morton et al. 2002). While deltas require increased sediment delivery to keep pace with accelerated compaction and sea level rise, the sediment fluxes reaching most major deltas have decreased due to construction of dams and reservoirs in the upstream river network (Vörösmarty et al. 2003). The increased rates of RSLR associated with these anthropogenic factors lead to increased coastal flood risk in deltas (Tessler et al. 2015a, b).

**Fig. 2:**
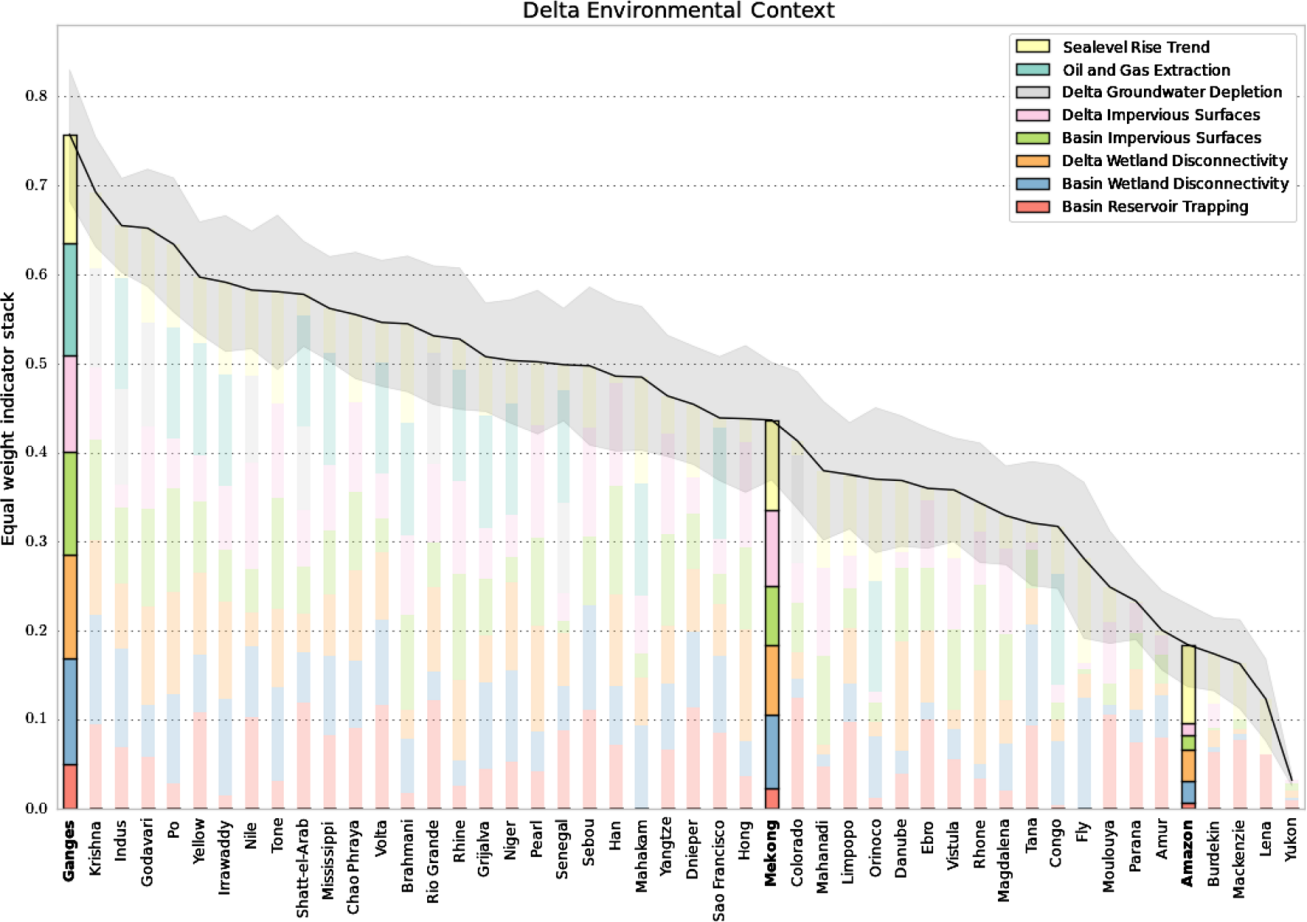
Selected rank-normalized anthropogenic impacts on deltas associated with sea level rise and land subsidence. The three focus deltas of this study are highlighted in the context of 48 global deltas. The *gray* region indicates the uncertainty of each delta’s aggregate anthropogenic impact following Tessler et al. (2015a, b)

Following the method of Tessler et al. (2015a, b), we have estimated the relative magnitude of eight anthropogenic drivers associated with relative sea level rise in deltas (Fig. 2). From the coastal delta domain, the anthropogenic drivers include hydrocarbon extraction, groundwater extraction, impervious surface area, and wetland disconnectivity. From the upstream contributing watershed, we include impervious surface area, wetland disconnectivity and sediment trapping in artificial reservoirs. We also include estimates of sea level rise trends from the oceanic domain. For global consistency, these indicators are derived from global-scale remote-sensing and numerical modeling. Each indicator is rank-normalized across 48 globally-distributed deltas, then aggregated to provide a relative scale for comparing the environmental state of river deltas and each delta’s susceptibility to RSLR. Constructed at the global scale, these estimates do not consider spatial patterns of environmental change within deltas, or important anthropogenic drivers of change other than those associated with RSLR, such as soil salinization, wetland loss and coastal eutrophication.

The Ganges–Brahmaputra, Mekong and Amazon deltas represent three different regions of the environmental stress space (Tessler et al. 2016). By these measures, the Ganges–Brahmaputra delta is the most environmentally stressed delta system in the study, with high levels for nearly all the environmental indicators. The only moderate-valued indicator is sediment retention in upstream reservoirs, though this may change in the near future with several new dams on the Brahmaputra River under construction or planned in India, Nepal and China (Zarfl et al. 2015). The Mekong delta, though affected by similar drivers as the Ganges–Brahmaputra delta, has an overall moderate level of environmental impact. New dam construction on the Mekong River is likely to be important in the near future (Kuenzer et al. 2015). There appears to be very low anthropogenic impact on the Amazon delta, relative to other deltas, with the largest stress associated with sea level rise.

## Feedbacks between population dynamics and environmental change

**Fig. 3:**
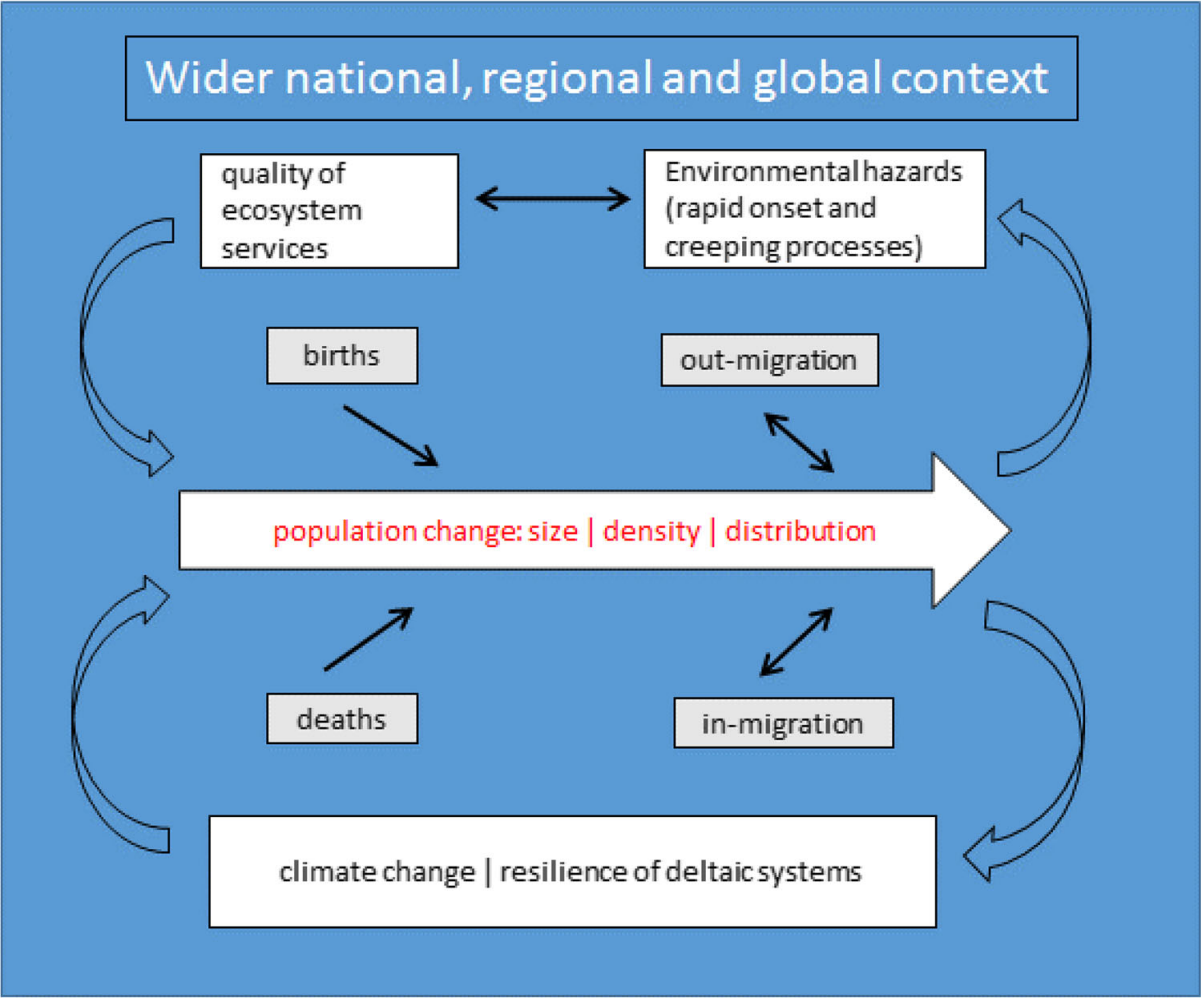
Feedbacks between population dynamics and environment

A delta-specific conceptual framework was developed (Fig. 3), which will serve for analyzing distinct components of population change in the three study deltas and how these demographic factors relate to environmental context in each delta. The framework will also serve to draw conclusions regarding the key demographic and environmental factors in each delta, and implications for development of sustainable management policies. All delta regions, as will be shown in the next sections of this paper, experienced relatively rapid population growth accompanied by decline in fertility and high out-migration. Demographic change can be explained at least partially, by the environmental context in which populations find themselves. The associations between population and environment have become key factors contributing to the international development debate (de Sherbinin et al. 2007; Hummel et al. 2012). Rapid population growth and resulting population density put pressure on provisioning ecosystem services in coastal areas, and the interactions between these factors are likely to intensify in the context of population growth and climate change (Kirwan and Megonigal 2013).

Specific components of demographic change can also be influenced by the quality of the natural environment, environmental hazards and creeping processes, such as salinity intrusion and arsenic contamination of water and soil resources. Environmental migration contributes to population loss in an area and might even lead to population collapse in certain areas of delta regions, as abandonment of settlements becomes the main coping strategy of vulnerable households (McLeman 2011). Mortality rates can be affected by the quality of provisioning ecosystem services, natural hazards and extreme weather events. For example, in Bangladesh, 3406 people died as a consequence of Cyclone Sidr in 2007 (Paul 2007). Deteriorating ecosystem services, such as poor water quality was found to be positively associated with health outcomes and child and maternal mortality (Brown et al. 2013; Cheng et al. 2012; Silva 2011). Salinization of soil and water has negative effects of quality of water and food security (Szabo et al. 2015a, b, c), which can lead to ill health. Finally, the quality of the environment can also have an impact on fertility rates, although this relationship is more complex and not fully established. There is, however, some evidence that environmental pollutants can negatively affect fertility, although research findings are not consistent (Fisch et al. 2003; Foster et al. 2008).

## Population growth and structure

**Fig. 4:**
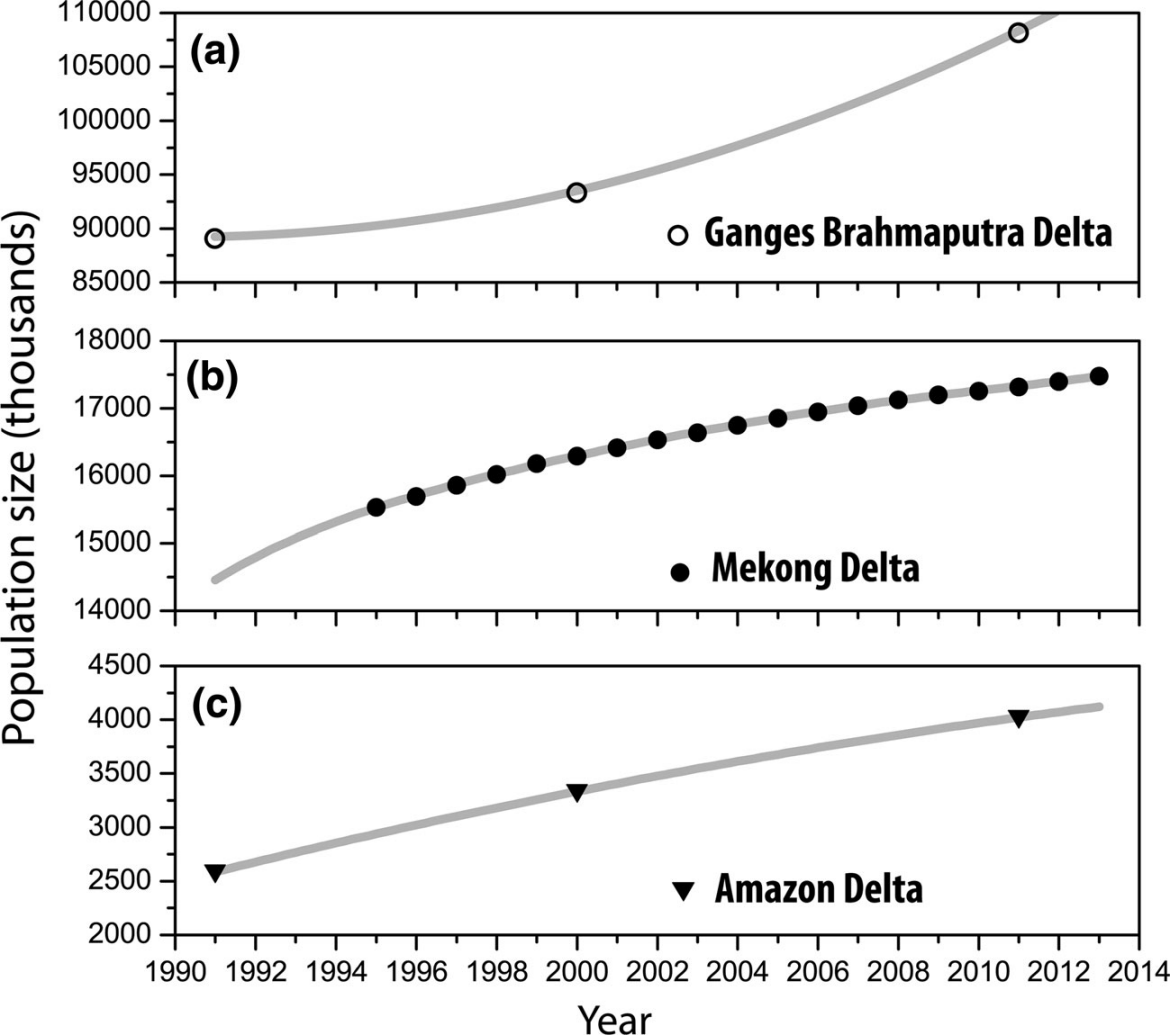
Recent population growth in the **a** Ganges–Brahmaputra, **b** Mekong and **c** Amazon deltas Data sources: Bangladesh Bureau of Statistics (BBS), Brazilian Institute of Geography and Statistics (IBGE), and General Statistics Office (GSO), Vietnam

Figure 4 illustrates the trends in population growth in the three delta regions. In the GBD, the population size in 2011 increased by approximately 19 million people compared to 1991, which represents a 17.5 % increase in population size over the last two decades (Fig. 4a). Based on the 2011 census data, the total population of the study area was about 108 million, comprising approximately 75 % Bangladesh’s population. Out-migration combined with below replacement level fertility rates in some districts contributes to changing population structure and negative rates of population growth. For example, during the last two decades, in Pirojpur district in south-western Bangladesh, the population decreased by around 18.4 %, while in the nearby Barisal district, the population declined by approximately 14.3 % (BBS 2012). Similarly to the GBD, in the past two decades, the Mekong delta continued to experience population growth despite falling fertility and relatively high out-migration (Dun 2011). The overall population in the Mekong delta increased from around 15.5 million in 1995 to over 17.5 million in 2013 (Fig. 4b). However, the Mekong delta is one of the regions with the lowest population growth rate in Vietnam with large spatial differences within the delta (Garschagen et al. 2012).

Likewise, the Amazon delta region has experienced continuous population growth since 1990 (Fig. 4c), most of it being in urban areas of the delta. As highlighted previously, the study area for the Amazon delta is shared by two states in the Northern region of Brazil. To the north, the state of Amapá includes nine municipalities, while to the south, the state of Pará includes 41 municipalities within the Amazon delta region. With approximately 4 million people, the population size of the Amazon delta is the smallest of the three delta regions. Since 1990, the region has experienced over 56 % increase in total population growth reaching a total of ca. 4 million people in 2010 (IBGE 2010). The majority of the population in 2010 (78.5 %) declared urban residency. However, there exists considerable variation within the region (IBGE 2010). The Amazon delta experienced slight decrease in rural population from 1990 to 2000, followed by a slight increase from 2000 to 2010. The increase in rural population reflects the increasing economic importance of forest and agroforestry products in the region, opening economic opportunities in rural areas and motivating strong connections between rural and urban areas (Brondizio et al. 2013).

**Fig. 5:**
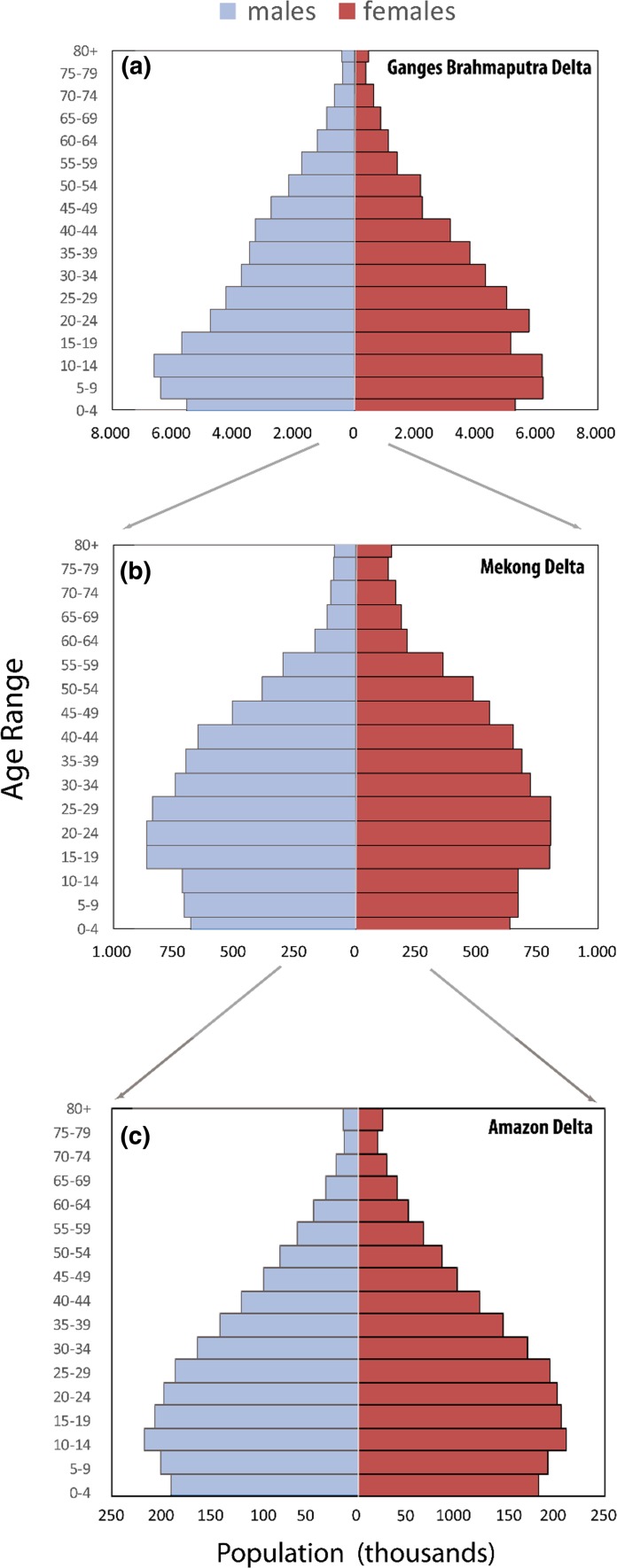
Population structure in the **a** Ganges–Brahmaputra (2011), **b** Mekong (2009) and **c** Amazon (2010) deltas Data sources: BBS, IBGE and GSO

When analysing population dynamics of delta regions, it is important to consider their population structures. The current population structure of the GBD (Fig. 5a) is the youngest, although it is visible that the youngest age groups (0–4 and 5–9) are disproportionally small, which reflects recent trends in fertility decline.  In the Mekong delta region, the population aged 15 to 35 constitutes the greatest percentage of the total population, while the bottom of the pyramid is relatively narrow indicating an aging population structure (Fig. 5b). This population structure is reflected in the region’s dependency ratios, which is around 42.3 % as compared to the national average of 44.7 % (General Statistics Office 2011a). Compared to the Mekong delta region, the population structure in the Amazon delta (Fig. 5c)  has not yet reached the rapid ageing pattern; however, its overall population is older than that of the GBD.

## Components of population change

### Mortality

**Fig. 6:**
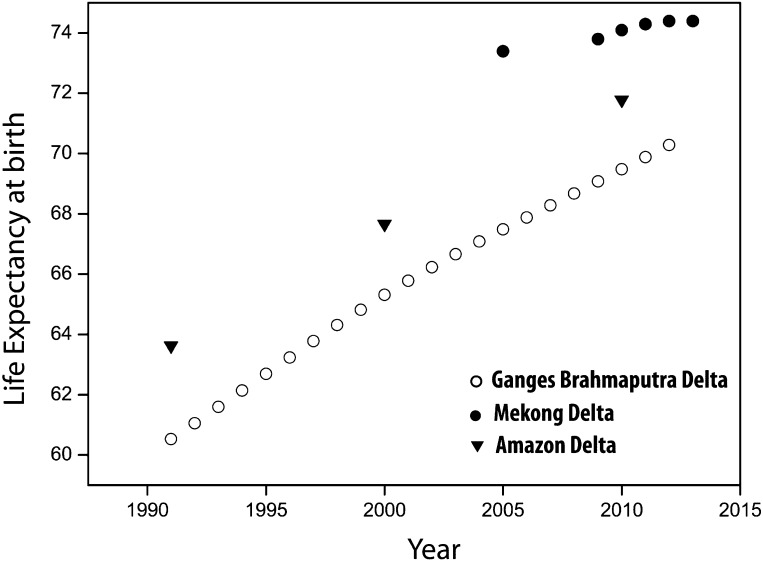
Recent trends in life expectancy at birth in the Mekong, Ganges–Brahmaputra and Amazon delta regions Data sources: World Development Indicators (WDI), IBGE and GSO, Vietnam. For GBD national data were used to approximate trends

Mortality is one of the components of population change. In Vietnam, life expectancy was 59.1 years in 1960 (World Bank 2015), and increased to 72.2 years in 2005 and 73.1 years in 2013 (General Statistics Office 2014). In the Mekong delta region, life expectancy increased from 73.4 years in 2005 to 74.4 years in 2013 (General Statistics Office 2014) (Fig. 6). Concerning child mortality, infant mortality rate (IMR) in the Mekong delta region is the second lowest regionally in Vietnam (after the Southeast region). The IMR in the Mekong delta is estimated at 12.0 per 1000 live births, while the national average is 15.3 per 1000 live births (General Statistics Office 2013). It is difficult to examine to what extent mortality trends have been affected by environmental factors. It is, however, reasonable to assume that gains in life expectancy could have been greater in the absence of environmental and climate change. The negative effects on population can occur through at least two sets of pathways: first, through direct impact of natural hazards on human life, and second, through the effects of the quality of environment, such as poor water quality linked to salinity ingression and arsenic contamination. As discussed in detail by the 2015 Lancet Commission on Health and Climate Change, the environmental impacts on human health, and thus, mortality rates are likely to be exacerbated by climate change. These will involve not only a potentially greater magnitude of natural hazard and diseases and creeping processes, such as soil and water salinization, but also the negative effects of higher temperatures (Watts et al. 2015). Also, it should be remembered that traditional socio-economic factors, such as access to quality healthcare and availability of and access to nutritious food have been found to be important determinants and mitigating factors of child, adolescent and adult health (Braveman et al. 2011).

With regards to the direct interlinkages between environmental factors and mortality (as conceptualized in Fig. 3), between 2001 and 2010, natural disasters affecting the country were responsible for death of 9.5 × 10^3^ people (Government of Vietnam 2011). Between 2000 and 2002, 1144 people were killed due to floods in the Mekong delta region (Central Committee for Flood and Storm Control 2015). Children were found to be particularly vulnerable to floods, especially in poorer households where parents worked outside often leaving children without supervision (Nguyen and James 2013). Secondly, indirect environmental effects on mortality rates include the quality of water and sanitation, which are associated with water-borne diseases, such as cholera and typhoid and paratyphoid fevers. Water quality in the frequently populated smaller waterways of the Mekong delta is relatively poor, regardless of the sources exploited (surface-, ground-water, rain water, piped water), with contamination by pesticides (Chau et al. 2015; Toan et al. 2013), nutrients, metals, salinity and microbial organisms (Wilbers et al. 2014a, b; Wilbers et al. 2013; Wilbers et al. 2014a, b). In peri-urban and rural regions of the Mekong delta, large portions of the population are directly exposed to polluted water and consume polluted water with treatments that do not eliminate all contaminants (Wilbers et al. 2014a, b; Wilbers et al. 2013; Wilbers et al. 2014a, b).

Similarly, in the GBD, environmental factors and climate change can have an important direct and indirect effect on human mortality. It should be stressed that Bangladesh as a country has achieved a significant progress in a number of health indicators, despite its relatively poor economic situation (Chowdhury et al. 2013). Current life expectancy in Bangladesh is estimated at 69 years for males and 71 years for females, an increase by 9 and 12 years, respectively since 1990 (WHO 2014) (Fig. 6). At the same time, however, maternal and child mortality rates are worryingly high. Maternal mortality ratio is 170 per 1,000,000 live births, which is similar to that of Pakistan and Cambodia and considerably higher than the maternal mortality rate (MMR) in Vietnam and Brazil (World Bank 2015). The population of the GBD is particularly vulnerable to cyclones, especially in coastal areas. It has been estimated that during the last 50 years, approximately 718 × 10^3^ people died due to cyclones (Haque et al. 2012). However, the death toll has fallen dramatically, and in 2007, 4234 people died as a result of cyclones compared to 500 × 10^3^ deaths in 1970 (Haque et al. 2012). This reflects improved early warnings and provision of a network of cyclone shelters. In addition to their direct effect, i.e., the loss of human life, cyclones affect the quality of water and increase the risk of disease transmission, in particular, in resource-poor areas. They can also have a post-disaster impact on mental health by increasing the risk of stress and depression (Haque et al. 2012; Shultz et al. 2005).

Finally, the population of the Amazon delta region experienced a rapid increase in life expectancy reaching 71.8 years in 2010, an increase by 7.2 years since 1991 (IPEA 2010) (Fig. 6). Overall, life expectancy is slightly higher in the Amapá state, although trends between both states (Pará and Amapá) are quite similar (IPEA 2010). Infant mortality rate remains high (21 per 1000) although declined more than twofold from 49.7 in 1991. This regional IMR is considerably higher when compared to the national IMR, which has been estimated at 15 in 2010 (World Bank 2015). According to a recent study by the International Institute for Environment and Development (IIED) (Viana et al. 2014), the infant mortality rate in Amapá state is the highest in Brazil. In the Brazilian Amazon region, natural disasters, in particular, floods, are associated with environmental and health impacts, including loss of life, however, disaggregated numbers are difficult to obtain (de Resende Londe et al. 2014). In this region, health problems have also been associated with the fast pace of urbanization accompanied by poor infrastructure. Health challenges included infectious diseases, such as malaria, in particular, in peripheral areas bordering forests (OPA 2010). Perhaps, the largest impact of environmental change can be noted in the case of migration.

### Migration

Migration is a key element of population change in all three study deltas, with the high environmental vulnerability of tropical deltas potentially being an important factor. At the same time, due to its unpredictability, migration is also the most difficult part of demographic modeling. Nonetheless, out-migration from three deltas as well as internal migration to the deltas’ megacities are likely to continue given unequal spatial development, biophysical transformations and environmental vulnerability (Seto 2011).The Mekong delta region is exposed to environmental hazards with extreme weather events leading to frequent flooding which affects people’s livelihoods (Dun 2011; Nguyen and James 2013). Also, slow onset hazards such as salinity intrusion continue to pose a severe risk to water and soil quality, and thus, to water supply and agriculture. Climate change is likely to exacerbate the existing risks, and thus, further affect future population distribution. Out-migration from the environmentally vulnerable areas is a widely recognized coping strategy (Rayhan 2008). Seasonal migration to the cities can provide income during the times of distress. For example, in the Thаnh Mÿ Tây commune in An Giang province, there were 5000 seasonal migrants reported in 2009 (Nguyen and James 2013). Between April 2012 and April 2013, there were a total of 121,443 out migrants from the Mekong delta region, with most of them moving to the Southeast region where Ho Chi Minh City is located (General Statistics Office 2013). As per the above-mentioned report, the net migration rates in the Mekong delta region are the same for males and females. Within the Mekong delta region, Bac Liêu Province reported the highest out-migration rates (−14.2 for males and −13.5 for females) (General Statistics Office 2013).

Similar to migration trends in the Mekong delta region, internal migration in the Bangladeshi GBD is an important demographic and social phenomenon. Environmental shocks combined with the economic vulnerability of large strata of the society are the key push factors affecting relatively high out-migration rates in coastal districts. A recent report by UNDP pointed out that 40 out of 64 districts have been identified to be environmentally at risk (Marshall and Rahman 2013), and the delta as a whole is the most environmentally stressed of those we analyzed (Fig. 1). High out-migration, in particular, from rural locations contributes to creation of large slum areas and informal settlements in cities. Because of limited or absent income generating opportunities, migration to cities is often perceived as a coping strategy for the rural poor. Also, crop losses or damage caused by natural hazards further exacerbate the existing social vulnerabilities. In Bangladesh, the number of life time migrants increased from 950 × 10^3^ in 1950 to 12,773 × 10^3^ in 2004, and out-migration from rural to urban areas grew from 7.3 per 1000 in 1984 to 25.9 per 1000 in 2010 (BBS 2011b). As is the case widely in other delta regions, climate change, and in particular, sea level rise, are projected to have negative impact on households’ livelihoods, which is likely to increase the volume of out-migration in the future (De Souza et al. 2015; Mallick and Vogt 2012).

Migration dynamics in the Amazon delta show interesting patterns. Based on the analysis of the data for the period 2001–2007 originating from the National Household Survey (PNAD), the state of Pará has consistently experienced high levels of out-migration. In contrast, migration patterns in Amapá have been highly volatile with only some years showing net out-migration flows (Ferreira-Filho and Horridge 2010). Nevertheless, recent research reports that over 28 % of the current population in Amapá originates from outside of this state which may explain the rising trends in population growth (Viana et al. 2014). Another body of research highlights that in the Amazon delta, similar to other delta regions, migration dynamics are largely intertwined with urbanization. The difficulty of categorizing and quantifying these migration trends is related to the fact that a large proportion of migration includes circular movements (Padoch et al. 2008).

### Fertility

**Fig. 7:**
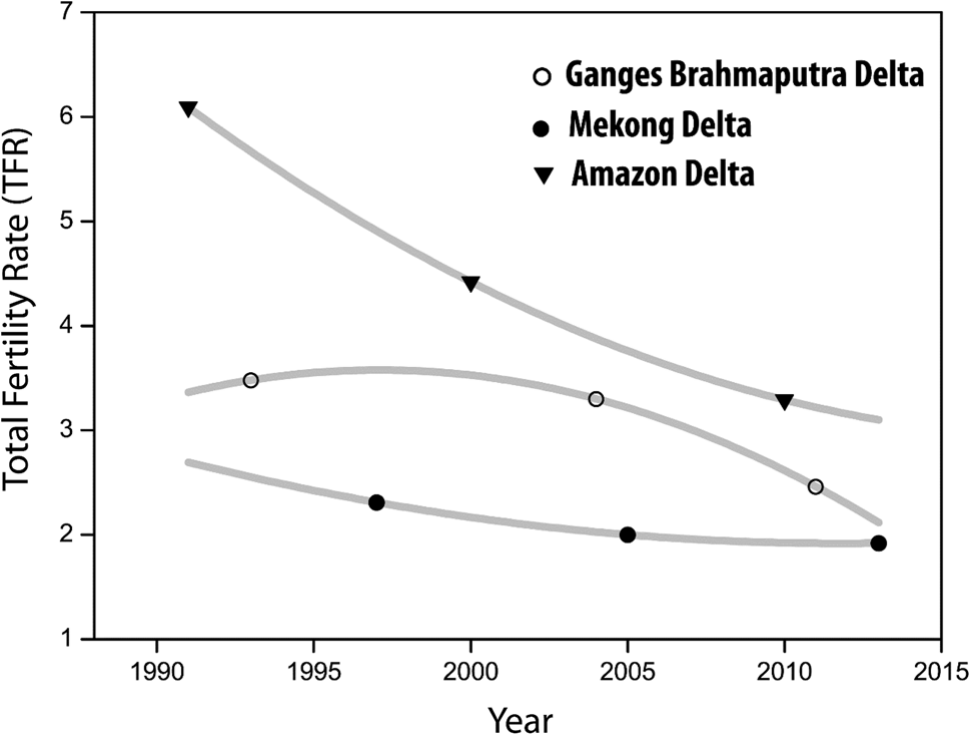
Recent trends in total fertility rate (TFR) in the Mekong, Ganges–Brahmaputra and Amazon delta regions Data sources: BBS, IBGE and General Statistics Office (GSO), Vietnam

As highlighted previously, during the past half-century, Vietnam has undergone rapid demographic transition with the TFR declining from 6.4 children per woman in 1960 to 1.8 children per woman in 2013 (World Bank 2015). Today, the unmet need for contraception is estimated at 4.3 % at the national level and 3.6 % for the Mekong delta region (General Statistics Office 2011b). Based on DHS data, it can be observed that since the 1990s, the TFR in the Mekong delta region has been continuously decreasing and is currently estimated at 1.92 (Fig. 7). In terms of early childbearing, the percentage of women aged 20–27 who had their first birth before the age of 18 is 5.8 % in the Mekong delta region, while the equivalent national proportion is 3 % (General Statistics Office 2011b).

Similar to the Mekong delta, analyzing trends in the total fertility rate in the GBD reveals a decline from 3.5 children per woman in 1993 to roughly below 2.5 in 2011 (Fig. 7). Fertility decline has been more pronounced for women from wealthier households than for women from poorer households. The average total fertility rates in the coastal divisions of Khulna and Barisal are below the national average except for Barisal district. The lowest fertility rates among the coastal districts are observed in Satkhira and Barguna districts with TFR 1.56 and 1.59, respectively (BBS 2012). Finally, since the early 1990s, the Amazon delta region has experienced the most rapid decline in TFR with a drop in TFR from over 6.1 children per women in 1991 to 3.3 children per woman in 2010. This fast decline in fertility mirrors the general trends in Brazil which is explained by a combination of government programs, migration from rural to urban areas, and social-cultural change regarding women’s rights and household roles (Siqueira et al. 2007). This trend started first in the urban areas of South and South East regions and then spread gradually to the rest of the country (Siqueira et al. 2007). A key feature of the process was the reliance on irreversible methods of contraception, such as female sterilization. In 1996, in North-eastern states, 51 % of married women between 15 and 49 reported having undergone sterilization (Caetano 2001), a trend confirmed for areas of the Northern region by Siqueira et al. (2007).

**Fig. 8:**
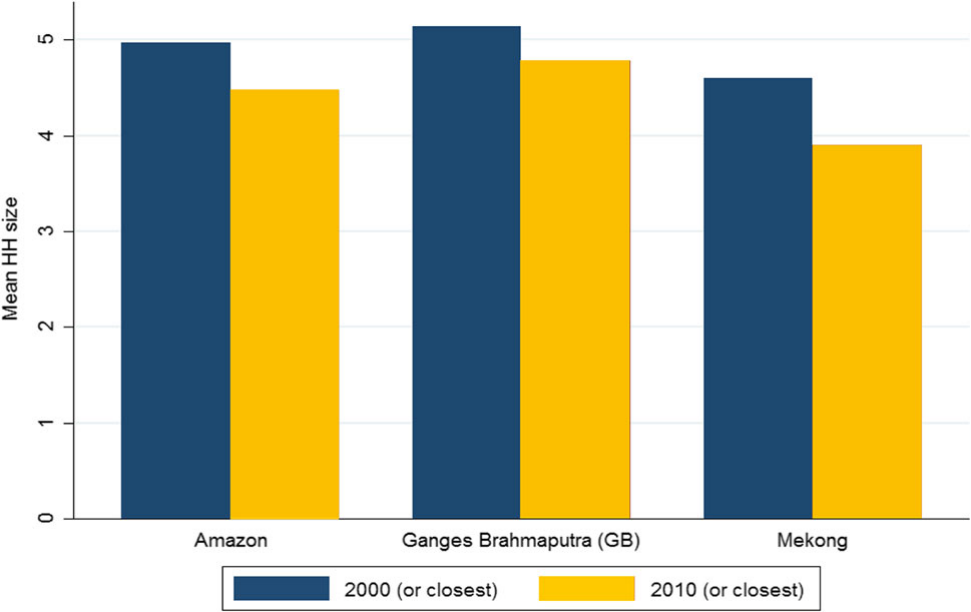
Change in mean household size across the three study areas Data sources: BBS, IBGE and GSO, Vietnam

These past trends indicate that future delta populations are likely to continue to significantly change and see smaller households with a greater proportion of elderly dependents. This predicted change is expected to be most rapid in the Mekong delta. Vietnam saw a decrease from an average household size of 4.8 in 1989 to 3.8 in 2009 (General Statistics Office 2011a), and in 2009, household size in the Mekong delta was estimated at 3.9 (Fig. 8). The trends toward smaller households are accompanied by a rising age at marriage and rising divorce rates. The ratios of divorce/separation to marriage are the highest in the Mekong delta and Southeast regions, which is likely to be associated with high rates of out-migration. Comparatively, in the GBD, there has been a trend toward smaller household size, although there exists considerable inter divisional differences. In our study area, an average household size varies from 4.3 in Khulna to 5.4 in Sylhet (BBS 2011b). It should be highlighted that during the last decade, all five divisions which fall under the study area have seen a decrease in household size. In 2001, the average family size in the delta region was 5.1, while in 2010 in was 4.8 (BBS 2011b). Finally, in the Amazon delta region, this decline was even more rapid, although an average household is larger than in the Mekong delta region. These changing population structures should be considered when assessing the consequences and implications of environmental change.

Conceptualizing direct links between environmental factors and fertility is more complex, albeit not impossible. As highlighted previously, an emerging body of evidence suggests that environmental pollutants may negatively affect fertility (Fisch et al. 2003; Foster 1998) and that the perceived risks related to climate change may be linked to couples’ fertility intensions (Arnocky et al. 2012). Also, fertility and family size can have direct and indirect effects on natural habitat and environmental and climate change at macro, meso and possibly micro levels. At the macro level, the most intuitive link is that through population growth, increased consumption and hence greater pollution and CO_2_ emissions. This link can operate at the local and regional level. For example, in the urban areas of GBD, including the megacity of Dhaka, rapid population growth has been associated with environmental degradation; including wetland vegetable cover reduction and fragmentation of landscape (Dewan et al. 2012). Given the changing fertility rates across the three study areas, shifts in population structure and projected impacts of climate change, it is crucial that further research is undertaken on the interlinkages between fertility and environmental factors in climate hotspots, such as delta regions.

## Discussion and conclusions

The increased population pressure on delta regions, together with the acceleration of urbanization and more intensive agricultural use, have magnified risks and exposure to relative sea level rise, flooding, and loss of ecosystem services. Furthermore, environmental factors exert a direct influence on population dynamics both over short (e.g. hurricanes and tropical storms) and mid/long (e.g. coastal retreat) term horizons. The complexity of the problem is compounded by the feedbacks between population and environmental factors in different socio-economic scenarios. O’Neill et al. (2015) described five basic Shared Socioeconomic Pathways (SSPs) to explore the possible outcomes in terms of mitigation and adaptation challenges related to climate change scenarios. The different narratives are based on alternative plausible futures of societal development at global scale and provide a set of qualitative descriptors of projected changes, including changes in demographic futures and environmental sustainability. However, extending the application of that general framework to specific areas, such as deltas, necessitates the identification of the distinctive factors (demographic, environmental, etc.) that are relevant regionally. Thus, this study aimed at investigating the dynamics of population change and identifying the key environmental factors expected to affect population dynamics across deltas, in general, and the three delta regions, in particular: the Ganges–Brahmaputra, the Mekong and the Amazon deltas (as defined in “Materials and methods”).

To examine the dynamics of population change across three delta regions, we analyzed the trends in population growth, population structure and three specific components of population change: fertility, mortality, and migration. The results of the analysis are summarized in Table 2, which provides an overview of key population dynamics, environmental factors and policy implications for each delta region. This summary draws from the conceptual framework presented in “Feedbacks between population dynamics and environmental change”. While there are considerable differences in terms of environmental and population change across the three deltas, several conclusions applicable to all three delta regions can be drawn. Firstly, our results suggest that, consistent with national averages, fertility rates in the delta regions have been steadily declining, falling below replacement levels in some geographical areas, such the Khulna and Barisal districts in south-western Bangladesh. This trend, combined with increasing life expectancy, improving child mortality rates and migration dynamics imply that in the longer term population in coastal delta regions are likely to stabilize or even decline, as recently projected for several districts in south-west Bangladesh (Szabo et al. 2015a, b, c). This differs from general expectations of expanding coastal populations and coastal cities (Neumann et al. 2015), and hence, is worthy of investigation in other deltas.

**Table 2:** Overview of important population change and environmental factors in each delta, and implications for development of sustainable management policies

Environmental threats that could affect demographic change	Timing/duration of impact	Population dynamics
Flooding^GB,M,A^ Sea level rise^GB,M,A^ Land subsidence^GB,M^ Cyclones and storm surge^GB,A(M)^ Soil salinization^GB^ Water salinization^GB,M,A^ Upstream impacts of climate change and dams/catchment management on river hydrology^GB,M,A^	Immediate—MT–LT LT MT–LT Immediate LT LT MT–LT	Population growth and size Population growth^GB,M,A^ Shifts in population structure (including population aging)^GB,M,A^
		Fertility Declining (sometimes below replacement) fertility^GB,M,A^
		Mortality Gains in life expectancy^GB,M,A^ Improvements in maternal and child health^GB,M,A^
		Migration High out-migration^GB,M^
		Socio-demographic change Declining household size^GB,M,A^ Rapid urbanisation^GB,M,A^
Policy implications		
Investments in rapid emergency response programmes and policies		
Social protection schemes in view of changing population structures, including population aging		
Policies aiming at tackling the drivers and consequences of changing and unequal population distribution		
Region specific rural development and urban planning policies tailored to local contexts		
Policies aimed at improving sanitation infrastructure and treatment particularly in urban areas		
Addressing potential labor shortages in the farming sector		
Investments in collection and analysis of desegregated demographic, socio-economic and environmental data		
Delta development plans		

*MT* medium term, *LT* long term, *GB* Ganges–Brahmaputra, *M* Mekong, *A* Amazon

Secondly, our findings suggest that all delta regions have been experiencing shifts in population structure resulting in aging populations, with the most rapid changes occurring in the Mekong delta. These expected shifts will complement the changes in population distribution. Although at different rates, rural–urban population distribution is likely to continue to change across the three deltas as rural families seek employment opportunities and better services in urban areas. Migration to urban areas, however, do not represent a rupture with the rural, but tend to be marked by intense circulation of people and resources, and thus, are likely to continue to shape the economy and governance of rural sectors in the three deltas. Finally, while environmental impacts on population trends and dynamics in the delta regions are hardly contestable, they remain difficult to quantify. Future research should therefore consider ways in which these integrated systems and associations could be analyzed and modeled (Lazar et al. 2015; Nicholls et al. 2013).

The results of the analysis presented in this study nevertheless have important policy implications. These delta regions should increasingly concentrate on addressing the needs of growing elderly populations and ensuring provision of care. This can be a challenging issue in developing countries where pension systems are often either weak, or non-existent. The changes in population structures and population distribution will also have implications in terms of labor supply. Coupled with out-migration, these trends will require the development of specific policies to tackle potential shifting from labor surplus to labor shortages in certain sectors and geographical areas. Additionally, environmental stressors, such as relative sea level rise, could entail large population displacements, including across national borders (Smajgl and Ward 2013). With an aging population, disaster preparedness programs will need to be appropriately adjusted to reflect the changing population needs during disasters.

Changes in occupational structure are also likely to be linked to internal migration and resulting urban growth. Addressing potential labor shortages in the farming sector is likely to be a challenge given that delta regions are food baskets for many nations (Foufoula-Georgiou et al. 2013), thus potentially contributing to reducing global food in security. Given the key role of demographic and environmental issues for sustainability of tropical deltas, as well as their wider interlinkages with other factors affecting sustainable development, such as public health and good governance, it is critical to undertake thorough assessments of specific population-environment dynamics on a case-by-case basis. Thus, locally informed SSP narratives, enriched by the specific characteristics of deltas, would improve the framework to assess mitigation and adaptation challenges.

The study presented here is unusual in looking at population dynamics across a set of deltas. More studies of this type are required to better characterize deltas and their populations around the world. In particular, focused research is urgently needed to further disentangle population–environment associations, through stakeholder engagement initiatives, targeted data collection, and dynamic/statistical modeling.

## References

[CR1] Abedin MA, Habiba U, Shaw R, Shaw R, Tran P (2012). Impacts of salinity, arsenic, and drought in Southwestern Bangladesh. Community, environment and disaster risk management.

[CR2] Arnocky S, Dupuis D, Stroink ML (2012). Environmental concern and fertility intentions among Canadian university students. Popul Environ.

[CR3] Balica SF, Wright NG, van der Meulen F (2012). A flood vulnerability index for coastal cities and its use in assessing climate change impacts. Nat Hazards.

[CR4] BBS (2011). Report of the household income and expenditure survey 2010.

[CR5] BBS (2011). Report on sample vital registration system—2010.

[CR6] BBS (2012). Community report. Barisal Zila.

[CR7] Brakenridge GR, Syvitski JPM, Overeem I, Higgins SA, Kettner AJ, Stewart-Moore JA, Westerhoff R (2013). Global mapping of storm surges and the assessment of coastal vulnerability. Nat Hazards.

[CR8] Braveman P, Egerter S, Williams DR (2011). The social determinants of health: coming of age. Annu Rev Public Health.

[CR9] Brondizio ES, Vogt N, Siqueira A, Morrison K, Hetch S, Padoch C (2013). Forest resources, city services: Globalization, household networks, and urbanization in the amazon estuary. The social life of forests.

[CR10] Brown SB, Nicholls RJ (2015) Subsidence and human influences in mega deltas: the case of the Ganges–Brahmaputra–Meghna. Sci Total Environ 527–528:362–374. doi:10.1016/j.scitotenv.2015.04.124 10.1016/j.scitotenv.2015.04.124 25974280

[CR11] Brown J, Cairncross S, Ensink JHJ (2013). Water, sanitation, hygiene and enteric infections in children. Arch Dis Child.

[CR12] Caetano AJ (2001) Fertility transition and the diffusion of female sterilization in Northeastern Brazil: the roles of medicine and politics. Paper presented at the Paper presented at the XXIV General Conference, IUSSP, Salvador, Brazil

[CR13] Central Committee for Flood and Storm Control (2015) Disaster database. http://www.ccfsc.gov.vn/KW6F2B34/Disaster-Database.aspx. Retrieved on 20 Dec 2015

[CR14] Chau ND, Sebesvari Z, Amelung W, Renaud FG (2015). Pesticide pollution of multiple drinking water sources in the Mekong Delta, Vietnam: evidence from two provinces. Environ Sci Pollut Res Int.

[CR15] Cheng JJ, Schuster-Wallace CJ, Watt S, Newbold BK, Mente A (2012). An ecological quantification of the relationships between water, sanitation and infant, child, and maternal mortality. Environ Health.

[CR16] Chowdhury AMR, Bhuiya A, Chowdhury ME, Rasheed S, Hussain Z, Chen LC (2013). The Bangladesh paradox: exceptional health achievement despite economic poverty. Lancet.

[CR17] Clarke D, Williams S, Jahiruddin M, Parks K, Salehin M (2015). Projections of on-farm salinity in coastal Bangladesh. Environ Sci Process Impacts.

[CR18] Dang LH, Li E, Nuberg I, Bruwer J (2014). Perceived risks of climate change and influencing factors: a study in the Mekong Delta Vietnam. Environ Manag.

[CR19] de Resende Londe L, Coutinho MP, Di Gregório LT, Lima Santos LB, Soriano E (2014). Water-related disasters in Brazil: perspectives and recommendations. Ambient Soc.

[CR20] de Sherbinin A, Carr D, Cassels S, Jiang L (2007). Population and environment. Ann Rev Environ Resour.

[CR21] De Souza K, Kituyi E, Harvey B, Leone M, Murali KS, Ford JD (2015). Vulnerability to climate change in three hot spots in Africa and Asia: key issues for policy-relevant adaptation and resilience-building research. Reg Environ Change.

[CR22] Dewan AM, Yamaguchi Y, Rahman MdZ (2012). Dynamics of land use/cover changes and the analysis of landscape fragmentation in Dhaka Metropolitan, Bangladesh. GeoJournal.

[CR23] Dun O (2011). Migration and Displacement triggered by floods in the Mekong Delta. Int Migr.

[CR24] Edmunds WM, Ahmed KM, Whitehead PG (2015). A review of arsenic and its impacts in groundwater of the Ganges–Brahmaputra–Meghna delta, Bangladesh. Environ Sci Process Impacts.

[CR25] Ericson JP, Vörösmarty CJ, Dingman SL, Ward LG, Meybeck M (2006). Effective sea-level rise and deltas: causes of change and human dimension implications. Glob Planet Change.

[CR26] Faisal IM, Parveen S (2004). Food security in the face of climate change, population growth, and resource constraints: implications for Bangladesh. Environ Manage.

[CR27] Ferreira-Filho JB, Horridge M (2010) Climate change impacts on agriculture and internal migrations in Brazil. Paper presented at the 13th Annual Conference on Global Economic Analysis, Penang, Malaysia. https://www.gtap.agecon.purdue.edu/resources/res_display.asp?RecordID=3293. Retrieved on 12 Dec 2015

[CR28] Fisch H, Andrews HF, Fisch KS, Golden R, Liberson G, Olsson CA (2003). The relationship of long term global temperature change and human fertility. Med Hypotheses.

[CR29] Foster KA (1998). Cities in our future: growth and form, environmental health and social equity. J Am Plan Assoc.

[CR30] Foster WG, Neal MS, Han MS, Dominguez MM (2008). Environmental contaminants and human infertility: hypothesis or cause for concern?. J Toxicol Environ Health Part B Crit Rev.

[CR31] Foufoula-Georgiou E, Overeem I, Saito Y, Dech S, Kuenzer C, Goodbred S, Harrison I, Anthony E, Brondizio E, Hutton J, Nicholls RJ, Matthews Z, Dearing J, Lazar A, Baschieri A, Newton A, Ramachandran R, Renaud FG, Sebesvari Z, Vörösmarty C, Tessler Z, Costa S, Ahmed KM, Rahman MM, Lintern G, Van Cappellen P, Durr H, Gao S, Marchand M, Bucx T, Nguyen VL, Goichot M, Paola C, Mohrig D, Twilley R (2013). A vision for a coordinated international effort on delta sustainability. Deltas Landforms Ecosyst Human Act.

[CR32] Garschagen M, Revilla Diez J, Kieu Nhan D, Kraas F, Renaud FG, Kuenzer C (2012). Socio-economic development in the Mekong Delta: between the prospects for progress and the realms of reality. The Mekong Delta system: interdisciplinary analyzes of a River Delta.

[CR33] General Statistics Office (2011). Age-sex structure and marital status of the population in Viet Nam: Vietnam population and housing census 2009.

[CR34] General Statistics Office (2011). Monitoring the situation of children and women—Viet Nam multiple indicator cluster survey 2011, final report.

[CR35] General Statistics Office (2013). The 1/4/2013 time—point population change and family planning survey—major findings.

[CR36] General Statistics Office (2014) Statistical data. http://www.gso.gov.vn/default_en.aspx?tabid=467&idmid=3. Retrieved on 20 Oct 2014

[CR101] Giri C, Ochieng E, Tieszen L, Zhu Z, Singh A, Loveland T, Masek J, Duke N (2011) Status and distribution of mangrove forests of the world using earth observation satellite data. Global Ecol Biogeogr 20(1):154–159. doi:10.1111/j.1466-8238.2010.00584.x

[CR37] Government of Vietnam (2011) National strategy on climate change.http://chinhphu.vn/portal/page/portal/English/strategies/strategiesdetails%3FcategoryId%3D30%26articleId%3D10051283. Retrieved on 19 Feb 2015

[CR38] Haque U, Hashizume M, Kolivras KN, Overgaard HJ, Das B, Yamamoto T (2012). Reduced death rates from cyclones in Bangladesh: what more needs to be done?. Bull World Health Organ.

[CR39] Higgins S, Overeem I, Tanaka A, Syvitski JPM (2013). Land subsidence at aquaculture facilities in the Yellow River delta, China. Geophys Res Lett.

[CR40] Hummel D, Adamo S, de Sherbinin A, Murphy L, Aggarwal L, Zulu L, Liu J, Knight K (2012). Inter- and transdisciplinary approaches to population-environment research for sustainability aims: a review and appraisal. Popul Environ.

[CR41] IBGE (2010) 2010 Census online. http://www.ibge.gov.br. Retrieved on 19 Feb 2015

[CR42] IPEA (2010) Instituto de Pesquisa Econômica Aplicada. http://www.ipea.gov.br/portal/. Retrieved on 19 Feb 2015

[CR43] Kay S, Caesar J, Wolf J, Bricheno L, Nicholls RJ, Saiful Islam AK, Haque A, Pardaens A, Lowe JA (2015). Modelling the increased frequency of extreme sea levels in the Ganges–Brahmaputra–Meghna delta due to sea level rise and other effects of climate change. Environ Sci Process Impacts.

[CR44] Kirwan ML, Megonigal JP (2013). Tidal wetland stability in the face of human impacts and sea-level rise. Nature.

[CR45] Kuenzer C, Campbell I, Roch M, Leinenkugel P, Tuan VQ, Dech S (2015). Understanding the impact of hydropower developments in the context of upstream-downstream relations in the Mekong river basin (vol 8, pg 565, 2013). Sustain Sci.

[CR46] Lazar AN, Clarke D, Adams H, Akanda AR, Szabo S, Nicholls RJ, Matthews Z, Begum D, Saleh AF, Abedin MA, Payo A, Streatfield PK, Hutton C, Mondal MS, Moslehuddin AZ (2015). Agricultural livelihoods in coastal Bangladesh under climate and environmental change—a model framework. Environ Sci Process Impacts.

[CR47] Lutz W, Prskawetz A, Sanderson WC (2002) Population and environment: methods of analysis, Population Council. Popul Dev Rev 28(A supplement to vol. 28)

[CR48] Mallick B, Vogt J (2012). Cyclone, coastal society and migration: empirical evidence from Bangladesh. Int Dev Plan Rev.

[CR50] Marshall R, Rahman S (2013). Internal migration in Bangladesh: character, drivers and policy issues.

[CR51] Mazzotti S, Lambert A, Van der Kooij M, Mainville A (2009). Impact of anthropogenic subsidence on relative sea-level rise in the Fraser River delta. Geology.

[CR52] McGranahan G, Balk D, Anderson B (2007). The rising tide: assessing the risks of climate change and human settlements in low elevation coastal zones. Environ Urban.

[CR53] McLeman RA (2011). Settlement abandonment in the context of global environmental change. Glob Environ Change Human Policy Dimens.

[CR54] MEF (2009). Bangladesh climate change action plan and strategy.

[CR55] Millennium Ecosystem Assessment (2005). Ecosystems and human well-being: synthesis.

[CR56] Mitra SN, Ali MN, Islam S, Cross AR, Saha T (1994). Bangladesh demographic and health survey, 1993–1994.

[CR57] Morton RA, Buster NA, Krohn D (2002) Subsurface controls on historical subsidence rates and associated wetland loss in southcentral Louisiana. Transact Gulf Coast Assoc Geol Soc 767–778. http://coastal.er.usgs.gov/gc-subsidence/gcags-paper/GCAGS02.pdf. Retrieved on 19 Feb 2015

[CR58] Neumann B, Vafeidis AT, Zimmermann J, Nicholls RJ (2015). Future coastal population growth and exposure to sea-level rise and coastal flooding—a global assessment. PLoS One.

[CR59] Nguyen KV, James H (2013). Measuring household resilience to floods: a case study in the Vietnamese Mekong river delta. Ecol Soc.

[CR60] Nicholls RJ (2004). Coastal flooding and wetland loss in the 21st century: changes under the SRES climate and socio-economic scenarios. Glob Environ Change Human Policy Dimens.

[CR61] Nicholls RJ (2011). Planning for the impacts of sea level rise. Oceanography.

[CR62] Nicholls RJ, Hutton CW, Lazar AN, Rahman MM, Salehin M, Ghosh T (2013) Understanding climate change and livelihoods in coastal Bangladesh. 2:40–42

[CR63] NIPORT, Mitra and Associates and ICF International (2013) Bangladesh Demographic and Health Survey 2011. Dhaka, Bangladesh and Calverton, USA

[CR64] NIPORT, Mitra and Associates and Macro International (2009) Bangladesh Demographic and Health Survey 2007. Dhaka, Bangladesh and Calverton, USA

[CR65] NIPORT, Mitra and Associates and ORCM (2001) Bangladesh Demographic and Health Survey 1999–2000. Dhaka, Bangladesh and Calverton, USA

[CR66] O’Neill BC, Kriegler E, Ebi KL, Kemp-Benedict E, Riahi K, Rothman DS, van Ruijven BJ, van Vuuren DP, Birkmann J, Kok K, Levy M, Solecki W (2015). The roads ahead: narratives for shared socioeconomic pathways describing world futures in the 21st century. Glob Environ Change.

[CR67] OPA (2010) Sustentabilidade ambiental e de saúde na Amazônia Legal, Brasil: uma análise através de indicadores. Brasília Organização Pan-Americana da Saúde (OPA)

[CR68] Padoch C, Brondizio E, Costa S, Pinedo-Vasquez M, Sears RR, Siqueira A (2008). Urban forest and rural cities: multi-sited households, consumption patterns, and forest resources in amazonia. Ecol Soc.

[CR102] Parry ML, Canziani OF, Palutikof JP, van der Linden PJ, Hanson CE (eds) (2007) Contribution of Working Group II to the Fourth Assessment Report of the Intergovernmental Panel on Climate Change. Cambridge University Press, Cambridge, UK, p 976

[CR69] Paul BK (2007). Why relatively fewer people died?. Nat Hazards.

[CR70] Rayhan I (2008) Assessing household vulnerability and coping strategies to floods: a comparative study of flooded and non-flooded areas in Bangladesh, 2005: Cuvillier Verlag

[CR71] Seto KC (2011). Exploring the dynamics of migration to mega-delta cities in Asia and Africa: contemporary drivers and future scenarios. Glob Environ Change.

[CR72] Shultz JM, Russell J, Espinel Z (2005). Epidemiology of tropical cyclones: the dynamics of disaster, disease, and development. Epidemiol Rev.

[CR73] Silva HP, Pinedo-Vasquez M, Ruffino ML, Padoch C, Brondízio ES (2011). Life is hard, life is beautiful: some perspectives on health and aging in amazonian rural populations. The Amazonian Várzea: the decade past and the decade ahead.

[CR74] Siqueira AD, D’Antona AO, D’Antona MF, Moran EF (2007). Embodied decisions: reversible and irreversible contraceptive methods among rural women in the Brazilian Amazon. Human Organ.

[CR75] Smajgl A, Ward J (2013). The water-food-energy nexus in the Mekong region: assessing development strategies considering cross-sectoral and transboundary impacts.

[CR76] Spencer T, Schuerch M, Nicholls RJ, Hinkel J, Lincke D, Vafeidis AT, Reef R, McFadden L, Brown S (2016). Global coastal wetland change under sea-level rise and related stresses: the DIVA Wetland Change Model. Glob Planet Change.

[CR77] Syvitski JPM, Saito Y (2007). Morphodynamics of deltas under the influence of humans. Glob Planet Change.

[CR103] Syvitski JPM, Kettner AJ, Overeem I, Hutton EWH, Hannon MT, Brakenridge GR, Day J, Vörösmarty C, Saito Y, Giosan L, Nicholls JR (2009). Sinking deltas due to human activities. Nat Geosci.

[CR78] Szabo S, Begum D, Ahmad S, Matthews Z, Steatfield PK (2015). Scenarios of population change in the coastal Ganges Brahmaputra Delta (2011–2051). Asia Pacific Popul J.

[CR79] Szabo S, Hossain S, Adger WN, Matthews Z, Ahmed S, Lazar A, Ahmad S (2015). Soil salinity, household wealth and food insecurity in tropical deltas: evidence from south-west coast of Bangladesh. Sustain Sci.

[CR80] Szabo S, Renaud FG, Hossain MdS, Sebesvari Z, Matthews Z, Foufoula-Georgiou E, Nicholls RJ (2015) Sustainable development goals offer new opportunities for tropical delta regions. Environ Sci Policy Sustain Develop 57(4):16–23. doi:10.1080/00139157.2015.1048142

[CR81] Tessler ZD, Vörösmarty CJ, Grossberg M, Gladkova I, Aizenman H, Syvitski JPM, Foufoula-Georgiou E (2015). Profiling risk and sustainability in coastal deltas of the world. Science.

[CR82] Tessler ZD, Vörösmarty CJ, Grossberg M, Gladkova I, Aizenman H (2015). A global empirical typology of anthropogenic drivers of environmental change in deltas. Sustain Sci.

[CR83] Toan PV, Sebesvari Z, Blasing M, Rosendahl I, Renaud FG (2013). Pesticide management and their residues in sediments and surface and drinking water in the Mekong Delta, Vietnam. Sci Total Environ.

[CR84] Tri VPD, Trung NH, Thanh VQ (2013). Vulnerability to flood in the Vietnamese Mekong Delta: mapping and uncertainty assessmen. J Environ Sci Eng.

[CR85] Viana V, Viana C, Euler A, Grieg-Gran M, Bass S (2014). Green economy in Amapá State, Brazil. Progress and perspectives.

[CR86] Vörösmarty CJ, Meybeck M, Fekete B, Sharma K, Green P, Syvitski JPM (2003). Anthropogenic sediment retention: major global impact from registered river impoundments. Glob Planet Change.

[CR87] Watts N, Adger WN, Agnolucci P, Blackstock A, Byass P, Cai WJ, Chaytor S, Colbourn T, Collins M, Cooper A, Cox PM, Depledge J, Drummond P, Ekins P, Galaz V, Grace D, Graham H, Grubb M, Haines A, Hamilton I, Hunter A, Jiang XJ, Li MX, Kelman I, Liang L, Lott M, Lowe R, Luo Y, Mace G, Maslin M, Nilsson M, Oreszczyn T, Pye S, Quinn T, Svensdotter M, Venevsky S, Warner K, Xu B, Yang J, Yin YY, Yu CQ, Zhang Q, Gong P, Montgomery H, Costello A (2015). Health and climate change: policy responses to protect public health. Lancet.

[CR88] WHO (2014) Global Health observatory data repository. http://gamapserver.who.int/gho/interactive_charts/mbd/life_expectancy/atlas.html. Retrieved on 30 May 2015

[CR89] Wilbers GJ, Sebesvari Z, Rechenburg A, Renaud FG (2013). Effects of local and spatial conditions on the quality of harvested rainwater in the Mekong Delta, Vietnam. Environmental Pollution.

[CR90] Wilbers GJ, Becker M, Nga LT, Sebesvari Z, Renaud FG (2014). Spatial and temporal variability of surface water pollution in the Mekong Delta, Vietnam. Sci Total Environ.

[CR91] Wilbers GJ, Sebesvari Z, Renaud FG (2014). Piped-water supplies in rural areas of the Mekong Delta, Vietnam: water quality and household perceptions. Water.

[CR92] Wong PP, Losada IJ, Gattuso JP, Hinkel J, Khattabi A, McInnes KL, Saito Y, Sallenger A (2014) Coastal systems and low-lying areas. In: Field CB, Barros VR, Dokken DJ, Mach KJ, Mastrandrea MD, Bilir TE, Chatterjee M, Ebi KL, Estrada YO, Genova RC, Girma B, Kissel ES, Levy AN, MacCracken S, Mastrandrea PR, White LL (eds) Climate change 2014: impacts, adaptation, and vulnerability. Part A: Global and Sectoral Aspects. Contribution of Working Group II to the Fifth Assessment Report of the Intergovernmental Panel on Climate Change (pp. 361–409). Cambridge University Press, Cambridge

[CR93] World Bank (2000) Bangladesh-climate change and sustainable development. World Bank http://documents.worldbank.org/curated/en/2000/12/1047483/bangladesh-climate-change-sustainabledevelopment. Accessed 20 Feb 2016

[CR94] World Bank (2015) The World development indicators. http://data.worldbank.org/indicator/all. Accessed 20 Oct 2015

[CR95] Zarfl C, Lumsdon AE, Berlekamp J, Tydecks L, Tockner K (2015). A global boom in hydropower dam construction. Aquat Sci.

